# ﻿*Thylacopterisminuta* (Polypodiaceae), a new fern species from Myanmar

**DOI:** 10.3897/phytokeys.199.83107

**Published:** 2022-06-09

**Authors:** Kiyotaka Hori, Phyo Kay Khine, Tao Fujiwara, Thant Shin, Harald Schneider

**Affiliations:** 1 The Kochi Prefectural Makino Botanical Garden 4200-6 Godaisan, Kochi 781-8125, Japan The Kochi Prefectural Makino Botanical Garden Kochi Japan; 2 Center for Integrative Conservation, Xishuangbanna Tropical Botanical Garden, Chinese Academy of Sciences, Menglun, Mengla, Yunnan 666303, China Xishuangbanna Tropical Botanical Garden, Chinese Academy of Sciences Mengla China; 3 Makino Herbarium, Tokyo Metropolitan University, 1-1 Minami-osawa, Hachioji, Tokyo 192-0397, Japan Tokyo Metropolitan University Tokyo Japan; 4 Forest Research Institute, Yezin, Nay Pyi Taw, Myanmar Forest Research Institute Yezin Myanmar

**Keywords:** Myanmar, new species, Polypodiaceae, *Thylacopteris*

## Abstract

The genus *Thylacopteris* is a small, phylogenetically isolated genus belonging to the fern family Polypodiaceae. This study describes a new species, *Thylacopterisminuta*, based on collections obtained during field surveys of Shan State, Myanmar. This new species is distinct from other species of *Thylacopteris* in its small size and presence of sclerenchyma strands in the rhizome. This species is also distinct from the only other species of *Thylacopteris* with molecular data available, *T.papillosa*, in a plastid *rbcL* phylogeny of Polypodiaceae. This new discovery of *Thylacopteris* from Myanmar suggests that this genus is still overlooked in Southeast Asia.

## ﻿Introduction

With about 1,652 species, Polypodiaceae is the second largest family of pteridophytes (ferns and lycophytes) (PPGI 2016). Species of this family share the presence of some characteristics such as yellowish or greenish spores, round or flat, yellowish-brown, exindusiate sori, and creeping rhizomes usually covered by scales (Zhang et al. 2013). Despite the progress in our understanding of the generic classification of these ferns as summarized in PPGI (2016), some issues are still unresolved. Based on recent molecular studies, Testo et al. (2019) segregated the genus *Bosmania* Testo, *Dendroconche* Copel. and *Zealandia* Testo from the paraphyletic genus *Microsorum*. Link, and Zhao et al. (2020) expanded the definition of the genus *Lepisorus* (J.Sm.) Ching. In addition, new species are still being discovered in Polypodiaceae in recent studies (Khine et al. 2016; Zhao et al. 2017) around Southeast Asia. To improve the pteridophyte flora of Southeast Asia, collections and molecular studies of Polypodiaceae are still needed in this area. In this study, we address the relationships of *Thylacopteris* specimens obtained in Shan State, Myanmar. The genus *Thylacopteris* includes currently only two species (Rödl-Linder 1994) of which one, *Thylacopterisdiaphana* (Brause) Copel., is endemic to New Guinea. The other species, *T.papillosa* (Blume) Kunze ex J.Sm, is known to occur throughout Malesia but has not been found in the north of the Isthmus of Kra (Rödl-Linder 1994).

The genus *Thylacopteris* Kunze ex J.Sm. was established by Smith (1875) with *T.papillosa* as the type species. This species was originally described as *Polypodiumpillosum* Blume based on an accession collected in Java (Blume 1828). As distinct characteristics of *Thylacopteris* from *Polypodium* sensu stricto, Blume (1828) mentioned the deeply sunken sori and the articulation of the lateral segments to the rachis. Subsequently, Copeland (1947) added a second species by introducing the combination *T.diaphana* (Brause) Copel. based on *P.diaphanum* Brause, which was based on an accession collected in New Guinea (Brause 1912). Some studies pointed out the close relationships of the genus *Thylacopteris* with either *Goniophlebium* or *Polypodium* (Christensen and Holttum 1934; Ching 1978; Tryon and Tryon 1982). Subsequently, *Thylacopteris* was treated as a group of *Polypodium* (Holttum 1966; Tryon and Lugardon 1991), or of uncertain systematic position in Polypodiaceae (Hennipman et al. 1990). Finally, utilizing DNA-based phylogenetics, *Thylacopteris* was found to be sister to a clade including *Goniophlebium*, *Lepisorus*, and *Microsorum* (Schneider et al. 2004). Considering the reported results of phylogenetic studies focusing on Polypodiacae, *Thylacotperis* was included in a broadly defined Microsoroideae in PPG I (2016). Reflecting its isolated phylogenetic position, Chen et al. (2019, 2021) introduced a tribe Thylacoptereae only including *Thylacopteris*.

In the recent years, some studies have reported new species (Khine et al. 2016) or new records of ferns (Nwe et al. 2016; Khine et al. 2017; Hori et al. 2019; Khine and Schneider 2020) from Myanmar. The discovery of new species and new records reflect the lack of comprehensive fern floristic studies of Myanmar (Khine et al. 2017; Khine and Schneider 2020), which limit the ability to report and manage the conservation of Myanmar’s unique biodiversity (Khine and Schneider 2020). Here, we describe a new species of the genus *Thylacopteris* from Myanmar and the first record of this genus in the country based on morphological characteristics and molecular phylogenetic analysis of Polypodiaceae.

## ﻿Materials and methods

We collected three specimens in Shan State, Myanmar during the inventories conducted under the leadership of the Makino Botanical Garden team on 13^th^ September 2015, 16^th^ September 2015, and 30^th^ September 2019 together with the team from the Xishuangbanna Tropical Botanical Garden, Chinese Academy of Sciences. To identify the new species *Thylacopterisminuta* sp. nov., the following characteristics were studied carefully and compared to the description and specimens of previously described species: the size and shape of plants, morphology of leaves including the shape of segments, stipe, anatomy of rhizome, scales, and the morphology of reproductive organs including sori, sporangia, and spores. Voucher specimens were deposited at the herbarium of the Kochi Prefectural Makino Botanical Garden (MBK), Xishuangbanna Tropical Botanical Garden (HITBC), Queen Sirikit Botanic Garden (QBG) and the Forest Research Institute, Myanmar (RAF). Herbarium codes follow Thiers (2021).

The plastid *rbcL* gene was employed for phylogenetic analysis. Total DNA was extracted from silica-dried leaves using cetyltrimethylammonium bromide (CTAB) solution according to the method of Doyle and Doyle (1990). PCR amplification was performed using the primers af3 and cr3 (Hori et al. 2018) and PrimeSTAR Max DNA Polymerase (Takara, Kyoto, Japan). PCR involved an initial denaturation step at 95 °C for 10 min, followed by 35 cycles of denaturation, annealing, and elongation steps at 98 °C for 10 s, 55 °C for 5 s, and 72 °C for 8 s, respectively (Model 9700 Thermal Cycler, Applied Biosystems, Foster City, CA, USA). The PCR products were purified using Illustra ExoStar 1-Step (GE Healthcare, Wisconsin, USA) and used as templates for direct sequencing. Reaction mixtures for sequencing were prepared using the BigDye Terminator v3.1 Cycle Sequencing Kit (Applied Biosystems, Foster City, CA, USA). The reaction mixtures were analyzed using an ABI 3130 Genetic Analyzer (Applied Biosystems, Foster City, CA, USA).

To estimate the phylogenetic position of the accession of interest, plastid *rbcL* sequences of Polypodiaceae were obtained from Genbank (https://www.ncbi.nlm.nih.gov/genbank/), covering all genera accepted in PPG I (2016) as far as data availability enabled. To reflect recent progress in our understanding of the natural classification of Polypodiaceae, the treatment of some taxa deviated from PPGI by adapting new concepts (Testo et al. 2019; Zhao et al. 2020; Chen et al. 2021). In the Genbank database, some accessions contained indels, which should not be present in *rbcL* since it is a protein-coding gene; we removed such low-quality accessions. The final data set included 94 accessions of Polypodiaceae, three samples of *Thylacopterisminuta* from Myanmar, and a set of outgroup taxa including *Davallia*, *Oleandra*, *Nephrolepis* and *Tectaria* (Table 1). The *rbcL* sequences were aligned using MUSCLE (Edgar 2004) and analyzed with Bayesian inference (BI) using MrBayes 3.2.6 (Ronquist et al. 2012) and maximum likelihood (ML) using MEGA X software (Kumar et al. 2018). Based on BIC values, GTR + G + I model was selected as the best-fit model of sequence evolution for BI analysis by jModelTest 2.1.10 (Darriba et al. 2012), and Tamura 3-parameter + G + I model was selected for the ML analysis by MEGA X software. Four chains of Markov chain Monte Carlo were run simultaneously and sampled every 100 generations for 1 million generations in total. Tracer 1.7.1 (Rambaut et al. 2018) was used to examine the posterior distribution of all parameters and their associated statistics, including estimated sample sizes. The first 2,500 sample trees from each run were discarded as burn-in. In ML analysis, initial trees for the heuristic search were obtained automatically by applying the Neighbor-Join and BioNJ algorithms to a matrix of pairwise distances estimated using the Tamura 3-parameter model, and then selecting the topology with superior log likelihood value. A discrete Gamma distribution was used to model evolutionary rate differences among sites (5 categories (+G, parameter = 0.9922)). The rate variation model allowed for some sites to be evolutionarily invariable ([+I], 57.20% sites). The tree was drawn to scale, with branch lengths measured in the number of substitutions per site. The bootstrap method with 1,000 replications was employed in ML analysis.

**Table 1.** Accessions of rbcL sequences in this study.

Accessions of rbcL sequences	Species
AF468205	*Adenophorusmontanus*
AY529147	*Aglaomorphaacuminata*
AF470349	*Aglaomorphacoronans*
AY529150	*Aglaomorphaheraclea*
MW138159	*Alansmiasmithii*
MT215977	*Archigrammitismarquesensis*
JQ685380	*Arthromerislehmannii*
MG948938	*Ascogrammitisanfractuosa*
EU482962	*Bosmaniamembranacea*
MT215995	*Calymmodoncucullatus*
MF318061	*Campyloneurumbrevifolium*
MF317971	*Campyloneurumlorentzii*
MF318013	*Campyloneurumrigidum*
MW138183	*Ceradeniakalbreyeri*
KM218797	*Chrysogrammitismusgraveana*
EF178615	*Cochlidiumserrulatum*
MT657584	*Ctenopterellablechnoides*
KM218775	*Dasygrammitisbrevivenosa*
MZ957125	*Davalliapulchra*
KM114198	*Davallia solida var. fejeensis*
MN018180	*Dendroconcheannabellae*
MN018176	*Dendroconchesayeri*
DQ227292	*Dictymiabrownii*
DQ164441	*Dictymiamckeei*
MW138254	*Enterosoratrifurcata*
KM218794	*Galactodeniaparrisiae*
MN017598	*Goniophlebiumamoenum*
AB043100	*Goniophlebiumformosanum*
DQ078627	*Goniophlebiummicrorhizoma*
AB043098	*Goniophlebiumniponicum*
MT657640	*Goniophlebiumpercussum*
AB043099	*Goniophlebiumpersicifolium*
MT657642	*Goniophlebiumsubauriculatum*
MT216033	*Grammitiscincta*
AB232409	*Gymnogrammitisdareiformis*
AF470322	*Lecanopteriscarnosa*
AF470329	*Lecanopteriscrustacea*
AF470325	*Lecanopterisluzonensis*
GU387043	*Lellingeriadissimulans*
MT169815	*Lepisoruscarnosus*
MN623364	*Lepisorushederaceus*
MT169813	*Lepisoruslongifolius*
MT169824	*Lepisorusnormalis*
AY362564	*Lepisorusnudus*
EU482971	*Lepisorussuperficialis*
GQ256304	*Lepisorusthunbergianus*
GQ256310	*Lepisorusuchiyamae*
MH768462	*Leptochilusdecurrens*
MH768470	*Leptochilusdigitatus*
MH768471	*Leptochilussaxicola*
GU376488	*Leucotrichummitchellae*
KF992501	*Loxogrammelanceolata*
DQ227294	*Loxogrammesalicifolia*
GU476898	*Melpomeneanazalea*
MW138194	*Microgrammalycopodioides*
AY362579	*Microgrammasquamulosa*
MF317960	*Microgrammavacciniifolia*
AY362344	*Micropolypodiumhyalinum*
LC496693	*Microsorumcuspidatum*
KY099830	*Microsorummembranifolium*
DQ179633	*Microsorumscolopendria*
MW620392	*Moranopteristaenifolia*
MT216066	*Nephrolepiscordifolia*
MT216068	*Nephrolepishirsutula*
EF463254	*Niphidiumcrassifolium*
MF317999	*Niphidiumlongifolium*
JQ904094	*Notogrammitisbillardierei*
EF463242	*Oleandraarticulata*
AB232405	*Oleandrapistillaris*
MT657589	*Oreogrammitisforbesiana*
EF463255	*Peclumaeurybasis*
AY362588	*Peclumaptilodon*
KT780748	*Peclumasicca*
MW138202	*Phlebodiumpseudoaureum*
MN623367	*Platyceriumbifurcatum*
AY362591	*Pleopeltisfructuosa*
EF463258	*Pleopeltissanctae*-*rosae*
KF909057	*Pleurosoriopsismakinoi*
KF909059	*Polypodiumscouleri*
KF186527	*Polypodiumvirginianum*
AB044899	*Polypodiumvulgare*
MT657600	*Prosaptiaalata*
EF463259	*Pyrrosiapolydactyla*
AY362558	*Pyrrosiarupestris*
EF463260	*Pyrrosiaserpens*
KM218771	*Radiogrammitisholttumii*
AY096199	*Selligueafeei*
AF470347	*Selligueahastata*
AY529171	*Selliguealaciniata*
MW138195	*Stenogrammitislimula*
DQ168808	*Synammiaintermedia*
KF667652	*Tectariagriffithii*
EF463274	*Tectariatrifoliata*
KM218802	*Terpsichoreaspleniifolia*
KM218758	*Themeliumdecrescens*
LC685475	*Thylacopterisminuta* sp.nov., Baba et al. 103191
LC685476	*Thylacopterisminuta* sp.nov., Baba et al. 103361
LC685054	*Thylacopterisminuta* sp.nov., Hori et al. 108601
AY459175	*Thylacopterispapillosa*
MH665089	*Thylacopterispapillosa*
KM218780	*Tomophyllummacrum*
MG452028	*Zealandiapowellii*
DQ401117	*Zealadiapustulatum*
DQ179635	*Zealandiavieillardii*
KM218793	*Zygophlebiadevoluta*

## ﻿Results and discussions

**Figure 1.**
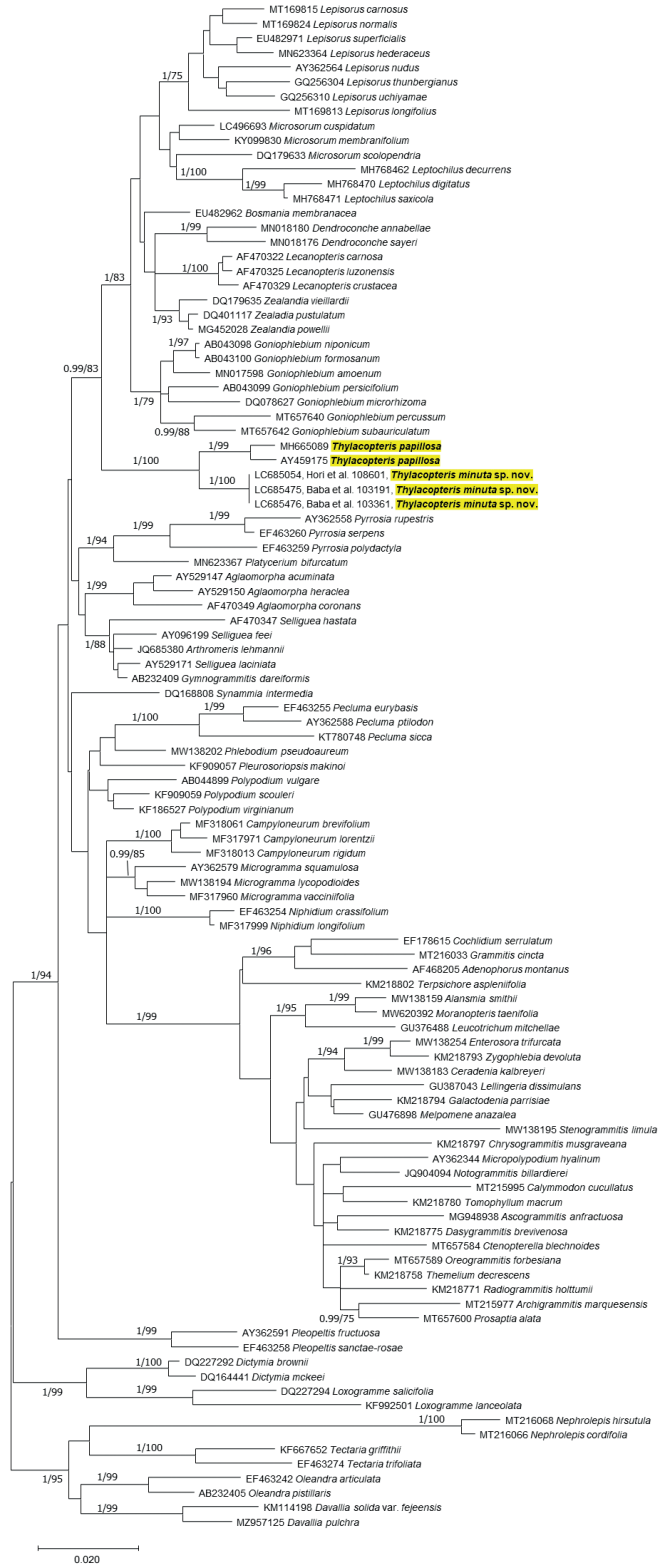
Phylogenetic hypothesis selected by applying maximum likelihood (the highest log-likelihood = −11294.22) to *rbcL* sequences. Posterior probabilities (> 0.90) and bootstrap percentages (> 70%) of Bayesian inference/maximum likelihood analyses are depicted at each node.

The aligned matrix included 1209 bp of *rbcL*, of which 329 bp (27%) were parsimony-informative. The ML (the highest log likelihood = −11294.22) tree showed that the three accessions of *Thylacopterisminuta* sp.nov (Baba et al. 103191, 103361, Hori et al. 108601) comprised a clade with two accessions of *T.papillosa* (Fig. 1). The *rbcL* sequence of Myanmar accessions of *Thylacopteris* had 25 substitutions relative to *T.papillosa*. The phylogenetic placement of *T.diaphana* is unresolved because DNA sequences were not available in this study. However, at least, morphological characteristics of the new species *T.minuta* can be differentiated from those of *T.diaphana* and *T.papillosa* as described below.

### ﻿Taxonomic treatment

#### ﻿ Thylacopteris minuta

Taxon classificationPlantaePolypodialesPolypodiaceae

K.Hori & Khine sp. nov.

2DE698DB-E278-5D06-907C-B7CA6C9AF0EA

urn:lsid:ipni.org:names:77299336-1

##### Diagnosis.

*Thylacopterisminuta* is similar to *T.papillosa* with 20–40 sclerenchyma strands per rhizome in cross-section. However, *T.minuta* is distinct from *T.papillosa* with sori shallowly sunken vs. *T.papillosa* sori deeply sunken. In addition, the lamina of *T.minuta* has a maximal length of 15 cm vs. a maximal length of 59 cm in *T.papillosa*. *Thylacopterisminuta* is distinct from the New Guinea endemic *T.diaphana*, which lacks sclerenchyma strands in the rhizome, has superficial sori, and lamina with a maximal length of 45 cm.

##### Type.

**Figure 2.**
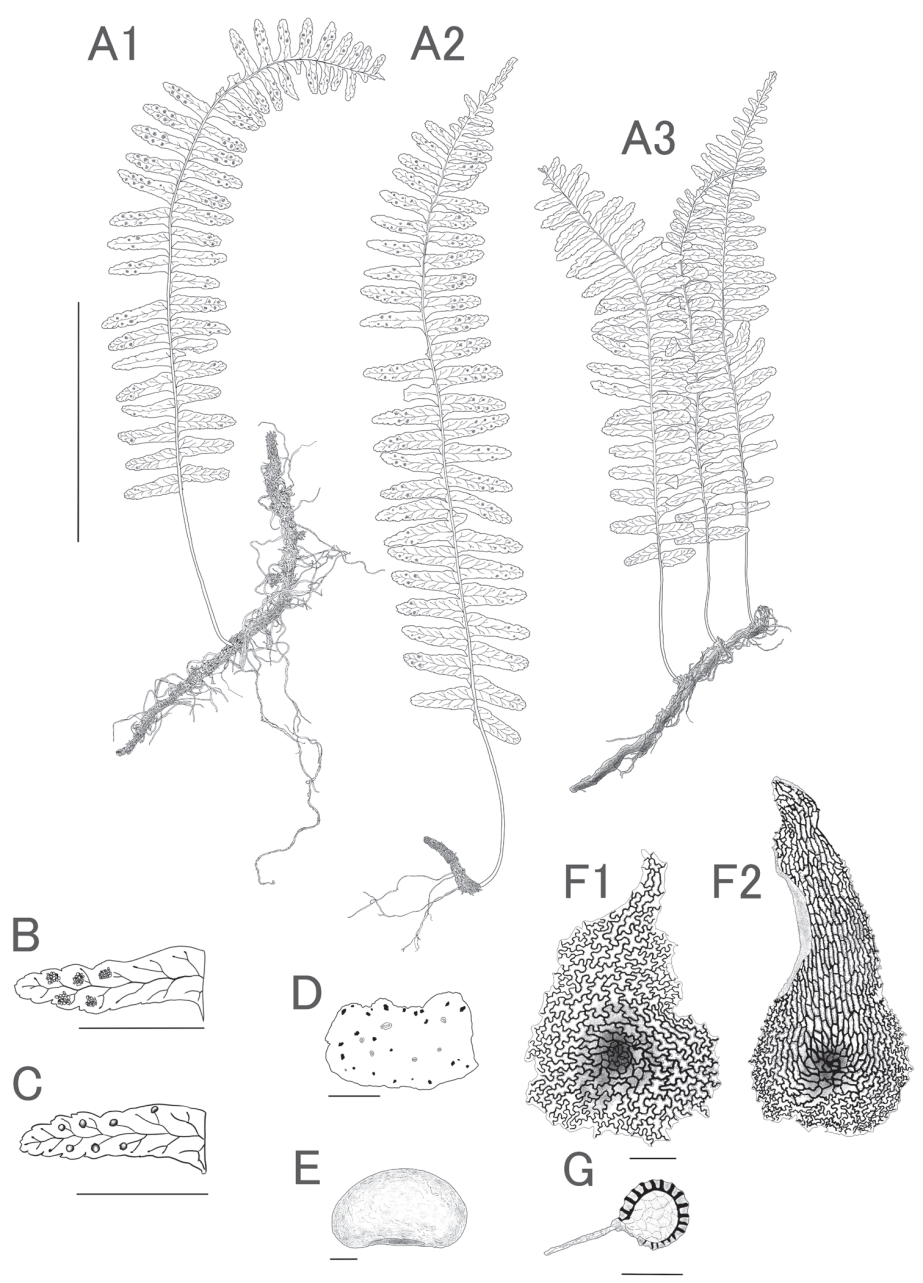
*Thylacopterisminuta* K. Hori & P.K.Khine (holotype, *Hori et al. 108601* = *MBK0328421*, illustration by K.Hori) **A** habit **B** abaxial view of a middle segment **C** adaxial view of a middle segment **D** cross-section of rhizome **E** spore **F** rhizome scales **G** sporangium. Scale bars: 5 cm (**A1–A3**); 1 cm (**B–C**); 0.5 mm (**D**); 10 µm (**E**); 20 µm (**F**); 200 µm (**G**).

**Figure 3.**
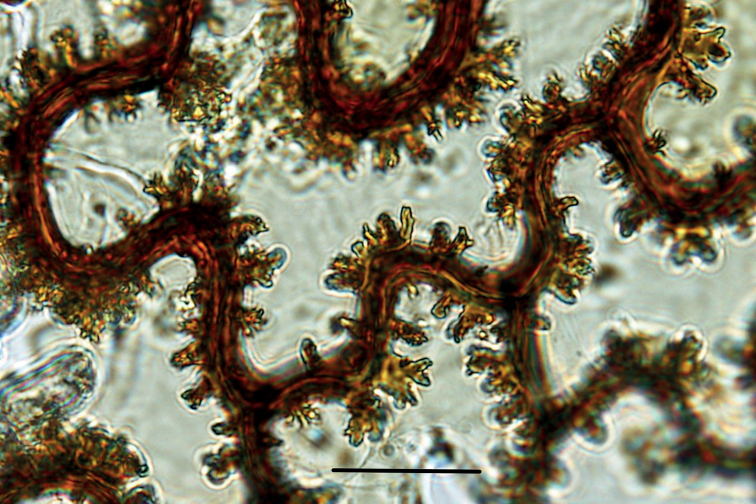
Warty, thickened cell walls of rhizome scales (*MBK 0328421*; photo taken using DP20 microscope camera, OLYMPUS, Japan). Scale bar: 2.5 µm.

**Figure 4.**
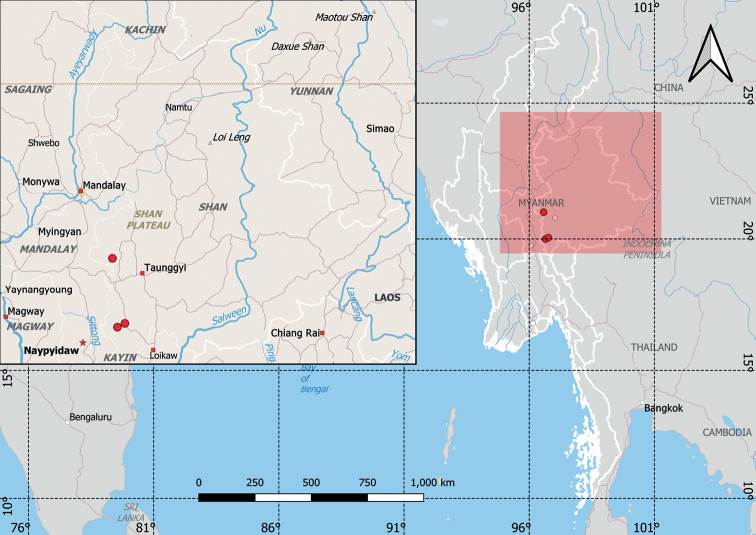
The distribution of *Thylacopterisminuta* sp.nov.

**Figure 5.**
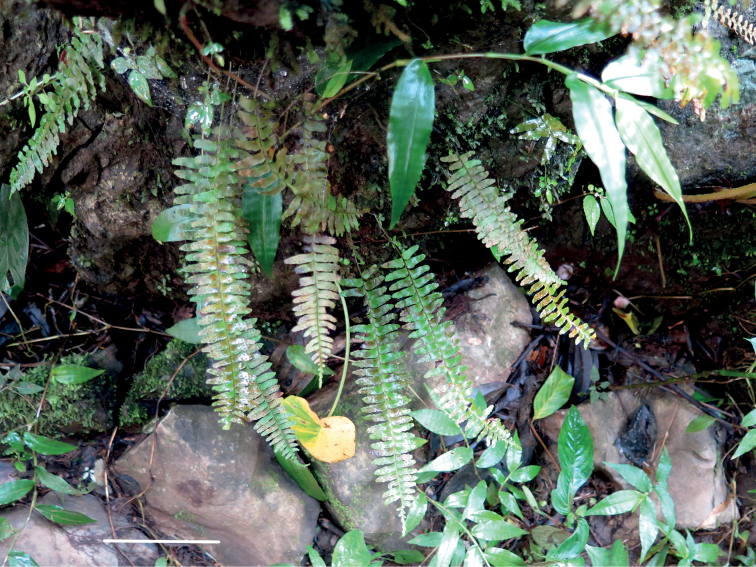
*Thylacopterisminuta* in its natural habitat (*MBK 0328421*). Scale bar: 5 cm. (Photo by P.K. Khine).

Myanmar: Shan State; Ah Lel Chaung reserve forest, Ywangan Township. 20°59'44.8"N, 96°34'26.81"E, ca.1325 m, 30 Sep. 2019, K. Hori, P.K. Khine [“Kine”], T. Fujiwara, M. Nagashima, P.P. Shwe & A.K. Moe 108601 (holotype: MBK 0328421 (herbarium barcode), Figs 2–5 isotype: HITBC, RAF).

##### Epilithic.

***Rhizome*** long-creeping, weakly branched, 1.0–2.0 mm in diam. (without scales), light brown, densely clothed with scales, phyllopodia sometimes prominent, these 1.0–2.0 mm high, 5.0–10.0 mm apart; 20–40 black sclerenchyma strands in rhizome, longitudinal, scattered in the ground tissue. ***Rhizome scales*** evenly inserted, dull brown, fragile, adpressed or apically spreading, quite densely set, deciduous, deltoid or ovate, 1.0–1.5 mm long, 0.5–1.0 mm wide, gradually narrowed from base to apex, sometimes with wavy margins, apex acute or rounded. ***Cell walls of rhizome scales*** dark brown, jigsaw-puzzle-shaped and wavy at basal and central part of scales, thickened, densely warty, in a single layer or double layers in basal scales. ***Fronds*** monomorphic, articulate to rhizome, petiolate. ***Stipes*** glabrous, 3.0–5.0 cm long, 0.7–1.0 mm diam, yellowish green. ***Blades*** membranous, lanceolate, 7.0–15.0 cm long, 2.0–4.0 cm wide, equally wide all along or rather wider above the basal part, pinnatisect, yellowish green. ***Segments*** glabrous, 20–30 pairs, lanceolate, ascending at an angle of 90°, 0.8–2.3 cm long, 0.3–0.5 cm wide, apically obtuse, entire at basal margin, crenate at apical margin, lower segments sometimes reduced, apical segments continuously reduced, terminal segments adnate or caudate. ***Veins*** free, once-forked, excurrent with terminal hydathodes. *Sori* exindusiate, uniserial on each side of costa, placed medially between costa and margin, shallowly sunken, 0.5–1.0 mm in diam., depth of papillae 0.2–0.5 mm, paraphyses absent. ***Sporangium*** globe-shaped, long stalked, 200–250 µm in diam., annulus vertical, indurated cells 10–13. ***Spores*** bilateral, oblong, light yellow, 40–60 µm long, 25–35 µm wide in lateral view, laesura 20–25 µm long, exospore smooth, perispore thin, surface shallowly wrinkled, globules absent.

##### Distribution.

Myanmar.

##### Habitat.

Epilithic, growing on shady surfaces of limestones (1–3 m high) in semi-evergreen or evergreen forest; altitude 940–1450 m.

##### Etymology.

The name refers to the relatively small size of this species compared to other species of *Thylacopteris*.

##### Additional specimens examined.

Myanmar: Shan State; Phaya Taung, Lein Le village, Paunglang Reserve Forest, Pinlaung Township; 19°59'41.0"N, 96°39'3.0"E, ca.947 m alt., 13 Sep. 2015, Y. Baba, K. Kertsawang, C. Kilgour, C. Puglisi, M. Rodda, P. Srisan­ga, T. Shin & P.P. Hnin 103191 (MBK0306471, duplicates on RAF, QBG). ibid., road between Nyaung Phyu village and Pinglaung village, Paunglang Reserve Forest, Pinlaung Township; 20°02'56.1"N, 96°46'00.1"E, ca.1448 m alt., 16 Sep. 2015, ibid., 103361 (MBK0313746, duplicates in RAF, QBG).

##### Notes.

The genus *Thylacopteris* is sometimes confused with *Goniophlebium* and *Polypodium* (Rödl-Linder 1994; Fraser-Jenkins 2020). Warty cell walls of rhizome scales (Fig. 3) can be used to conclusively identify the genus *Thylacopteris* (Rödl-Linder 1994).

### ﻿Key to species of the genus *Thylacopteris*

**Table:** 

1	Blades 7–15 cm long	***T.minuta***
–	Blades 30–60 cm long	**2**
2	Sori deeply sunken; sclerenchyma present in cross-section of rhizome	***T.papillosa***
–	Sori not sunken sclerenchyma; absent in cross-section of rhizome	***T.diaphana***

## Supplementary Material

XML Treatment for Thylacopteris minuta
